# Ventilation/Perfusion Scintigraphy in Children with Post-Infectious Bronchiolitis Obliterans: A Pilot Study

**DOI:** 10.1371/journal.pone.0098381

**Published:** 2014-05-22

**Authors:** Bo-Qia Xie, Wei Wang, Wen-Qian Zhang, Xin-Hua Guo, Min-Fu Yang, Li Wang, Zuo-Xiang He, Yue-Qin Tian

**Affiliations:** 1 Department of Cardiology, Chaoyang Hospital, Capital Medical University, Beijing, China; 2 Department of Respiration, Beijing Children's Hospital, Capital Medical University, Beijing, China; 3 Department of Thoracic surgery, Institute of Respiratory Disease, Chaoyang Hospital, Capital Medical University, Beijing, China; 4 Department of Nuclear Medicine, State Key Laboratory of Cardiovascular Disease, Fu Wai Hospital, National Center of Cardiovascular Disease, Chinese Academy of Medical Sciences and Peking Union Medical College, Beijing, China; 5 Department of Nuclear Medicine, Chaoyang Hospital, Capital Medical University, Beijing, China; The Hospital for Sick Children and The University of Toronto, Canada

## Abstract

**Purpose:**

Childhood post-infectious bronchiolitis obliterans (BO) is an infrequent lung disease leading to narrowing and/or complete obliteration of small airways. Ventilation and perfusion (V/Q) scan can provide both regional and global pulmonary information. However, only few retrospective researches investigating post-infectious BO involved V/Q scan, the clinical value of this method is unknown. This preliminary prospective study was aimed to evaluate the correlation of V/Q scan with disease severity, pulmonary function test results, and prognosis in children with post-infectious BO.

**Methods:**

Twenty-five post-infectious BO children (18 boys and 7 girls; mean age, 41 months) underwent V/Q scan and pulmonary function tests. Patients were followed after their inclusion. Ventilation index and perfusion index obtained from V/Q scan were used to measure pulmonary abnormalities. Spearman's rank correlation test of ventilation index and perfusion index on disease severity, lung function tests indices, and follow-up results were performed.

**Results:**

The median follow-up period was 4.6 years (range, 2.2 to 5.0 years). Ventilation index and perfusion index were both correlated with disease severity (*r* = 0.72, *p*<0.01 and *r* = 0.73, *p*<0.01), but only ventilation index was related to pulmonary function tests results (all *p*<0.05). In addition, Spearman test yielded significant correlations between perfusion index and prognosis (*r* = 0.77, *p*<0.01), and ventilation index and prognosis (*r* = 0.63, *p* = 0.01).

**Conclusions:**

For children with post-infectious BO, the present study preliminarily indicated that the degree of ventilation and perfusion abnormalities evaluated by V/Q scan may be used to assess disease severity, and may be predictive of patient's outcome.

## Introduction

Childhood post-infectious bronchiolitis obliterans (BO) is an infrequent chronic obstructive pulmonary disease due to profound inflammation that leads to narrowing and/or complete obliteration of the small airways [1]. In recent years, high-resolution computed tomography (HRCT) has been recognized as a useful tool for the diagnosis of BO because it can delineate the abnormalities in small airways, and has been used in the evaluation of the extent and progression of airway disease [2], [3]. However, whether or not the morphological changes detected by HRCT can be equated with function impairment in patients with BO is controversial [4]–[6]. As been reported by Donnelly et al [7], the changes on HRCT were only predictive of regional lung function in the most normal and severely diseased lung areas. On the other hand, of the conventional pulmonary function tests, spirometry is the most frequently used method, but this maneuver is difficult to apply in infants or young children as it requires patient cooperation. Although new techniques such as single occlusion technique and tidal breathing flow-volume curves have been developed to evaluate infants' lung function [8], [9], the assessment is time-consuming and the indices are not congruent with the conventional tests, thus making the comparison difficult even with a same child at a different age. Moreover, pulmonary function tests only provide overall lung function, the specific left/right and regional status could not be evaluated. As a non-invasive measurement, ventilation and perfusion (V/Q) scan can provide both regional and global information in patients with lung disease, and can offer additional functional information that was not provided by anatomical imaging. In addition, this technique is much easier to perform than that of function tests [10]. However, V/Q scan is underutilized in a pediatric population, and the indication of this method in post-infectious BO is not well established. Only few retrospective researches involved this method in the investigation of childhood BO disease [11], [12], and the clinical value of V/Q scan in post-infectious BO children is unknown due to the rare data. Therefore, the aim of this prospective study is to 1) evaluate the correlation of V/Q scan with disease severity; 2) assess the relationship of V/Q scan with pulmonary function test results; 3) explore the prognostic value of V/Q scan in children with post-infectious BO.

## Materials and Methods

Study protocol was approved by the Institutional Ethics Committee of Beijing Children's Hospital and Fu Wai Hospital. Children's parents signed informed consent before inclusion into the study.

### Study population

From June 2008 to August 2011, consecutive patients diagnosed of post-infectious BO at Beijing Children's Hospital were prospectively included in this study. The diagnosis of post-infectious BO was based on a typical clinical history followed by findings on chest radiography and thoracic HRCT that concurred with the diagnosis: 1) history of an acute and severe bronchiolitis/pneumonia; 2) recurrent cough, wheezing, respiratory distress after the acute event; 3) respiratory symptoms which are severe in disproportion to chest radiography findings; 4) Mosaic pattern and air trapping in HRCT; 5) exclusion of other chronic lung disease that progress with permanent respiratory symptoms [13], [14]. Patients were excluded if they had congenital heart disease, immunodeficiency or failed to accomplish V/Q scan. The interval between V/Q scan and pulmonary function tests was set as less than one week so as to minimize the change of lung function in these separate examinations. At admission, two expertized pediatric physicians evaluated the disease severity of each patient using an increasing score (0 =  none, 1 =  mild, 2 =  moderate, 3 =  severe) based on symptoms and lung physical signs, the evaluation method was described in Table 1. Disease severity reached consensus by the two physicians. Children's parents signed informed consent before inclusion into the study. Study protocol was approved by the Institutional Ethics Committee.

**Table 1:** Disease severity evaluation method.

Components of Severity	Score			
	0	1	2	3
**Cough**	None	Mild	Moderate	Severe
**Wheeze**	None	Intermittent	Persistent	Severe
**Exercise intolerance**	None	With strenuous activity	With mild activity	With rest
**Lung physical signs**	None	Occasionally	Persistent	Severe

Patient's disease severity was defined as none (summed score  = 0), mild (summed score ranged from 1 to 3), moderate (summed score ranged from 4 to 7), and severe (summed score ranged from 8 to 12).

### Pulmonary ventilation and perfusion scan

The dose activity of Tc-99m diethylenetriaminepentaacetic acid (DTPA) for ventilation scintigraphy was 0.4–0.6 MBq/kg (0.01–0.02 mCi/kg). Uncooperative children were sedated with oral chloral hydrate (40–60 mg/kg), and face mask was placed over the nose and mouth to enable the inhalation of Tc-99m DTPA aerosol. As for cooperative children, mouthpiece and nose clip were applied. The inhalation time was 5 minutes. A double-headed gamma camera equipped with low-energy, high-resolution, parallel-hole collimators (Infinia Hawkeye 4, GE, USA) was used to collect ventilation images at 8 views (posterior, right posterior oblique, right lateral, right anterior oblique, anterior, left anterior oblique, left lateral, and left posterior oblique). Acquisition parameters including a 128×128 matrix, a zoom of 1.0, and 300 kilo-counts per view.

Pulmonary perfusion scintigraphy was performed on the next day. Perfusion scan began with an intravenous injection of Tc-99m labeled macroaggregated albumin (MAA) (0.5–2.0 MBq/kg (0.01–0.05 mCi/kg) depending on children's age via a pre-inserted venous cannula. Perfusion images were acquired in the similar manner of ventilation scan except with 500 kilo-counts per view. All patients remained in supine position throughout the examination.

The dose of Tc-99m DTPA and Tc-99m MAA administered in this study were based on the “as low as reasonably achievable concept” and was consistent with the recently published Society of Nuclear Medicine guidelines [15].

Both ventilation and perfusion images were visually evaluated by 2 experienced nuclear physicians blinded to the clinical data. Based on 20-segment lung model [16], the apical segment and posterior segment in the left lung were combined as apicoposterior segment, and the anterior basal segment and medial basal segment in the right lung were combined as anteromedial basal segment, consequently, there were 18 segments in total. To quantify the abnormalities of ventilation and perfusion, we designed a ventilation index (VI) and a perfusion index (PI) using the following steps: first, each segment was scored on a 0 to 3 scale (0, normal; 1, mild reduction; 2, severe reduction; and 3, absent) for ventilation and perfusion respectively. The extent of abnormality was defined as 0.5 when the abnormal area is less than 50% of the affected lung segment, and was defined as 1.0 when the abnormal area is over 50%. When this was done, we calculated ventilation score and perfusion score for each lung segment by multiplying the severity score with the extent of abnormality. And then, VI/PI was acquired by dividing the sum of all ventilation scores/perfusion scores by 18. Consequently, the more the VI/PI deviated from 0, the worse the ventilation/perfusion was. The severity score and the extent of abnormality of each lung segment reached consensus between the two readers.

### Pulmonary function tests

For cooperative older children, spirometric parameters, including forced vital capacity (FVC), forced expiratory volume in 1 s (FEV_1_), and maximum mid-expiratory flow rate (MMEF) were measured according to the American Thoracic Society Guidelines, using a Jaeger-Masterlab (Erich Jaeger GmbH, Wuzburg, Germany). The spirometric parameters were expressed as percentages of predicated values and were expressed as percent of the references [17]. For infants and young children, tidal breathing flow-volume curves were analyzed by Master Screen Pad (Erich Jaeger GmbH, Wuzburg, Germany). Indices including tidal volume over body weight (VT/kg), ratio of time to peak tidal expiratory flow to total expiratory time (T_PEF_%T_E_), and ratio of volume to peak tidal expiratory flow to total expiratory volume (V_PEF_%V_E_) were calculated. Pulmonary compliance per kg weight (Crs/kg) and airway resistance (Rrs) were evaluated using single occlusion technique.

### Follow-up

Participants were regularly telephone followed by one investigator. Patients' prognostic status were scored using the same evaluation method of disease severity as displays in Table 1 (0 =  none, 1 =  mild, 2 =  moderate, 3 =  severe).

### Statistical analysis

Statistical analyses were carried out with SPSS (version 19.0, SPSS Inc). Data were described as frequencies, mean ± SD and medians with ranges. Spearman's rank correlation test was used to assess the strength of the relationship between variables. A probability value of <0.05 was considered statistically significant for all tests.

## Results

Thirty-two children diagnosed of post-infectious BO were initially recruited. Seven individuals were excluded because they did not accomplish V/Q scan. The remaining 25 children (18 boys and 7 girls; mean age ± SD, 41±40 months) constituted our patient population. The mean interval between pulmonary function test and V/Q scan was 4±2 days (range, 0–7 days).

### Patients' characteristics

**Figure 1:**
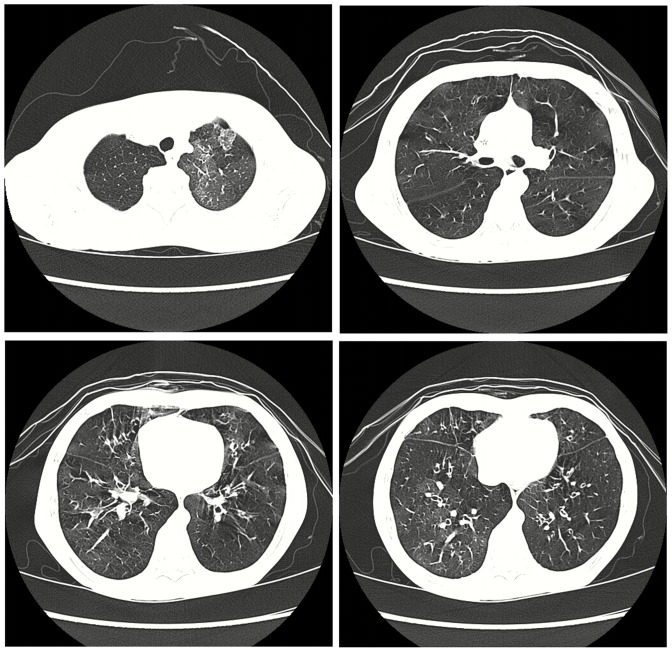
HRCT of patient No.-glass pattern with air trapping and bronchial thickening.

**Figure 2:**
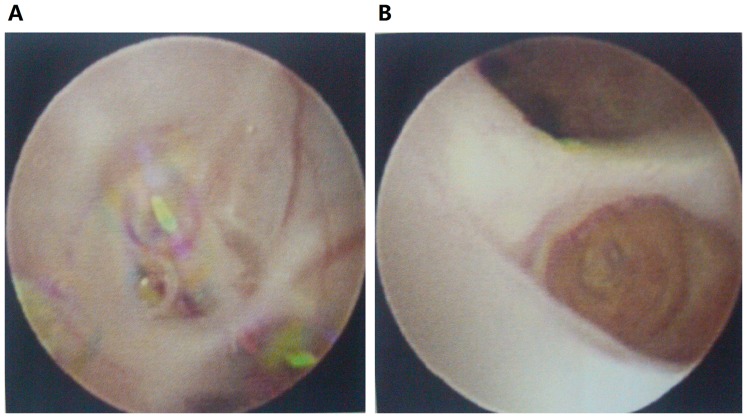
Fiberoptic bronchoscopy of the same patient shown in Figure 1. Complete obstructions were observed in the subsegmental anterior basal bronchus of the left lower lobe (A) and in the subsegmental lateral bronchus of the right middle lobe (B).

Table 2 presents the clinical and imaging findings of the 25 patients. Before been diagnosed as post-infectious BO, children had an initial lung insult at a median age of 16 months (0–142 months). Predisposing factors were identified as infections. During the initial episode, 22 patients (88%) required hospitalization. Of these individuals, 5 children developed heart failure and respiratory failure, 3 children had isolated heart failure, and one children had isolated respiratory failure. Mechanical ventilation was applied in 6 children. Cough and wheezing were the primary symptoms and persisted in all patients. Post-infectious BO was diagnosed at a median of 3 months (range, 1–43 months) after the initial lung insult. All children had mosaic pattern and air trapping by HRCT. Other radiological findings including bronchial wall thickening and bronchiectasis (Fig 1). At admission, disease severity was scored as 1 in nine children, 2 in seven children, and 3 in nine children (Table 2). Fiberoptic bronchoscopy was performed in 23 patients, yielding airway stenosis in 4 patients and complete obstruction in 8 patients (Fig 2). All children received supportive treatment, including inhaled corticosteroids and bronchodilators, antibiotics, and systemic corticosteroids during acute exacerbations. Twenty-three patients had bronchial lavage. Two patients needed mechanical ventilation. One patient needed intensive care management. The mean hospital stay was 42±20 days (range, 10–84 days).

**Table 2:** Clinical characteristics and imaging results of patients (n = 25).

Patient No.	Gender	Age (month)	Age at initial lung insult (month)	Symptoms	Chest X-ray	HRCT	PO_2_ (%)	PCO_2_ (%)	Disease severity	Days of hospitalization	VI	PI
**1**	Male	30	29	Nasal flaring, cyanosis, retractions, crackles	—	Be, consolidation	43	42	3	67	1.94	1.32
**2**	Male	52	11	—	—	BWT	69	39	1	31	1.50	1.03
**3**	Male	8	5	Crackles	Consolidation	Be, consolidation	65	40	2	33	1.38	0.74
**4**	Male	23	21	Crackles	—	BWT	84	42	2	14	0.29	1.18
**5**	Male	132	131	Nasal flaring, retractions	—	Be	83	34	2	30	1.47	0.21
**6**	Male	22	16	—	—		100	29	1	10	0.09	0.09
**7**	Female	10	7	—	—		74	29	1	44	1.29	0.35
**8**	Female	47	36	Fever, crackles	—	Consolidation	45	37	2	36	0.50	0.26
**9**	Female	88	84	Fever, crackles	Consolidation	Consolidation	75	30	2	58	1.12	0.88
**10**	Male	120	2	Crackles	—		57	32	1	26	0.44	0.85
**11**	Male	18	12	Crackles, retractions	Consolidation	Hyperinflation, consolidation	64	35	3	54	1.59	1.68
**12**	Male	67	24	Fever, crackles	Atelectasis	Be, consolidation,	65	32	2	55	1.88	1.88
**13**	Male	12	9	Crackles	—	Consolidation	74	39	2	37	1.06	0.38
**14**	Male	11	9	Fever, dyspnea, retractions, nasal flaring, crackles	Consolidation	Be, consolidation	60	36	3	84	2.15	2.15
**15**	Male	11	6	Fever	Consolidation	Consolidation	66	31	3	47	2.12	0.88
**16**	Female	19	1	Crackles	—		75	32	1	13	0.12	0.12
**17**	Female	60	1	Fever	Consolidation	BWT	62	35	3	59	2.18	2.18
**18**	Female	28	9	Crackles	—	Consolidation	73	36	2	43	1.41	1.41
**19**	Male	28	16	Crackles	Consolidation	Consolidation	77	36	1	31	1.71	1.06
**20**	Male	44	36	Fever, cyanosis, retractions	Consolidation	Consolidation	64	44	3	50	1.82	0.35
**21**	Male	108	107	Nasal flaring, cyanosis, retractions, crackles	Hyperinflation, consolidation	Consolidation	46	47	3	79	2.74	1.53
**22**	Female	84	83	Fever, crackles	Consolidation	Hyperinflation, consolidation	66	33	2	44	1.24	0.82
**23**	Male	144	142	Fever	Consolidation	Hyperinflation, consolidation	96	33	1	19	0.24	0.18
**24**	Male	10	8	Crackles	Consolidation	BWT	100	27	1	21	0.06	0.06
**25**	Male	32	21	Fever, crackles, retractions	Consolidation	BWT, consolidation	46	61	3	54	1.41	1.41

All patients had persistent cough and wheezing, so these manifestations were not listed in the symptoms column. All patients showed mosaic pattern and air trapping by HRCT, so these imaging findings were not listed in the HRCT column. BWT =  bronchial wall thickening; Be =  Bronchiectasis

### V/Q scan

**Figure 3:**
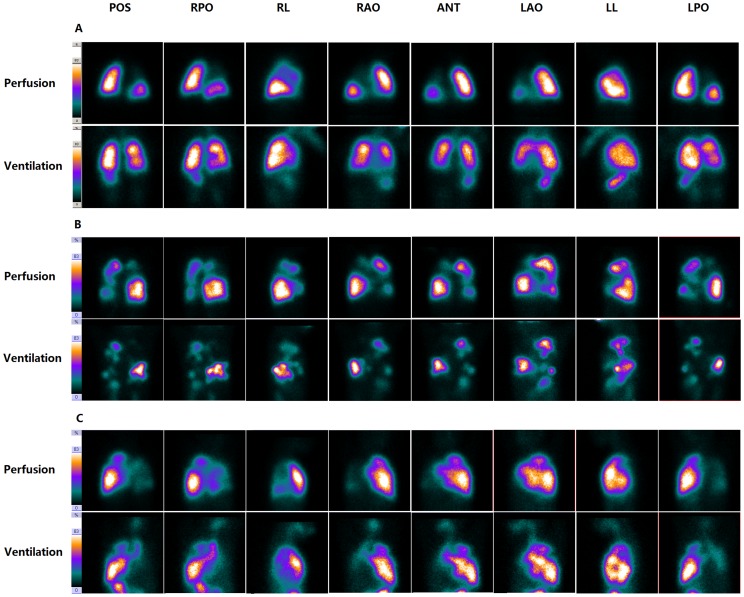
Three patterns of V/Q scan (A. mismatched ventilation; B. mismatched perfusion; C. matched ventilation-perfusion) in post-infectious BO children (patient No. 4, patient No. 21 and patient No. 12), displayed in 8 views. POS =  posterior, RPO =  right posterior oblique, RL =  right lateral, RAO =  right anterior oblique, ANT =  anterior, LAO =  left anterior oblique, LL =  left lateral, LPO =  left posterior oblique.

V/Q scan were performed at a median of 7 days (range, 1–34 days) after admission. Abnormal V/Q scan were detected in all patients, and three patterns of V/Q scan were observed in our patients: mismatched ventilation (better ventilation over perfusion) (Fig 3A), mismatched perfusion (better perfusion over ventilation) (Fig 3B), and matched ventilation-perfusion (concordant degree of the impairment of ventilation and perfusion) (Fig 3C). Of all 425 lung segments, 236 segments had impaired ventilation (56%) and 176 segments had reduced perfusion (41%). Comparing ventilation scan with perfusion scan, 176 segments were normal. In the remaining 249 segments, 125 segments had matched ventilation-perfusion, 99 segments had mismatched ventilation, and 25 segments had mismatched perfusion. The mean VI was 1.27±0.75, and the mean PI was 0.92±0.65.

Spearman test demonstrated significant correlations between disease severity and VI (*r* = 0.72, *p*<0.01) and PI (*r* = 0.62, *p*<0.01). Correlations were also yielded between the days of hospitalization and VI (*r* = 0.80, *p*<0.01) and PI (*r* = 0.73, *p*<0.01) (Table 3).

**Table 3:** Spearman's rank correlation test of VI and PI on disease severity, days of hospitalization, and lung function tests parameters.

Variable	VI		PI	
	R	P value	R	P value
**Disease severity**	0.72	0.00	0.62	0.00
**Days of hospitalization**	0.80	0.00	0.73	0.00
**FVC**	−0.94	0.01	−0.77	0.07
**FEV_1_**	−0.94	0.01	−0.49	0.33
**MMEF**	−0.77	0.07	−0.37	0.47
**T_PEF_%T_E_**	−0.63	0.02	−0.28	0.33
**V_PEF_%V_E_**	−0.57	0.03	−0.28	0.34
**Crs/kg**	−0.62	0.02	−0.49	0.08
**Rrs**	0.62	0.02	0.42	0.14

R =  Spearman's rank correlation coefficient

### Pulmonary function tests

Five children's pulmonary function tests data were excluded from the regression analysis because the interval between V/Q scan and pulmonary function test exceeded 1 week. Of the remaining 20 children, 6 cooperative older children had lung function measured by spirometry (Table 4) and 14 patients had their pulmonary function assessed by tidal breathing flow-volume curves and single occlusion technique (Table 5). Results from the above tests revealed increased resistance and decreased compliance in all patients, 4 patients had response to bronchodilator.

**Table 4:** Pulmonary function results in cooperative patients.

Patient No.	FVC*	FEV_1_ *	MMEF*	Response to bronchodilator
**5**	42.1	26.8	10.3	Negative
**9**	78.8	84.7	70.0	Negative
**12**	41.7	30.6	12.8	Negative
**21**	29.0	24.6	12.9	Negative
**22**	79.8	72.9	57.6	Negative
**23**	89.8	93.5	79.8	Positive
**mean±SD**	60.2±25.5	55.5±31.6	40.6±32.1	

*Values given as percent of predicated values.

**Table 5:** Pulmonary function results in infants and young children.

Patient No.	VT/kg (ml/kg)	T_PEF_%T_E_ (%)	V_PEF_%V_E_ (%)	Crs/kg (ml/kPa/kg)	Rrs (kPa*s/L)	Response to bronchodilator
**1**	8.5	10.7	12.7	4.9	6.0	Negative
**3**	7.7	7.9	14.0	6.9	6.5	Positive
**4**	8.4	19.8	26.2	8.1	4.0	Negative
**6**	10.8	16.2	20.5	11.9	3.7	Negative
**7**	8.3	14.7	17.5	6.3	5.5	Negative
**11**	9.5	13.2	16.6	7.6	5.4	Negative
**13**	8.6	10.4	11.1	9.9	5.8	Negative
**14**	7.7	11.5	17.1	7.0	5.8	Positive
**15**	9.3	10.3	13.9	9.2	5.2	Negative
**16**	8.0	17.9	21.3	10.6	3.4	Negative
**18**	9.2	17.3	21.2	8.0	5.6	Negative
**19**	6.1	12.2	17.3	3.6	6.2	Negative
**24**	7.9	21.2	25.9	9.7	2.1	Positive
**25**	9.8	11.8	16.5	7.0	5.1	Negative
**mean±SD**	8.6±1.1	13.9±4.0	18.0±4.6	7.9±2.2	5.0±1.2	

Spearman test demonstrated that FVC, FEV1, T_PEF_%T_E_, V_PEF_%V_E_, Crs/kg, and Rrs were correlated with VI (*r* = −0.94, −0.94, −0.63, −0.57, −0.62, and −0.62; all *p*<0.05). Contrarily, no pulmonary function test indices were correlated with PI (all *p*>0.05) (Table 3).

### Follow-up

Eight patients were lost during the follow-up period. The median follow-up period was 4.6 years (range, 2.2 to 5.0 years) from the diagnosis of post-infectious BO. At the end of follow-up, 2 patients became symptom-free, 7 patients had infrequent mild cough and preferable general well-being, and 8 patients had persistent cough and wheezing and decreased exercise tolerance. Of the 8 patients with continuous symptoms, 5 individuals required readmissions due to exacerbations of respiratory symptoms. Accordingly, the prognostic score was 0 in two children, 1 in seven children, 2 in three children, and 3 in five children.

Spearman test yielded significant correlations between prognostic score and VI (*r* = 0.63, *p* = 0.01) and PI (*r* = 0.77, *p*<0.01).

## Discussion

To the best of our knowledge, this preliminary study is the first prospective research exploring the correlation of V/Q scan with disease severity and pulmonary function test results, and to investigate the prognostic value of V/Q scan in children with post-infectious BO. Our results showed that better ventilation was correlated with less severe disease and better lung function test results, and better perfusion was related to a promising prognosis.

Results from the current study showed that VI is significantly correlated with that of pulmonary function test results, severity of disease, and days of hospitalization. This was an expected result since ventilation scan has been established as an accurate method for evaluating airway disease [10], [18]. The present study suggested that ventilation scan maybe applicable in evaluating post-infectious BO children's lung function. Besides, as ventilation scan can be performed for children in different age groups, this technique may be applied as a convenient objective tool to provide pulmonary function results regardless of children's age. Contrary to VI, no pulmonary function test results were found to be correlated with PI, this may be explained by the fact that perfusion is affected secondary to that of ventilation in post-infectious BO children, thus was not a sensitive tool for the direct measurement of airway impairment. Despite the insignificant relationship between PI and lung function test results, significant correlations were yielded between PI and disease severity and hospital stay. And this finding was congruent with a recently published study [11], which suggested that the number of hypoperfused segments in BO patients was correlated with the days of hospitalization. We speculate that the preserved blood supply to the lung is a prerequisite for effective treatment [19], [20], like oxygen therapy to relieve hypoxemia, or antibiotics for superimposed infections. Besides, hypoperfused lung does not function in pulmonary gas exchange.

Another interesting finding from the current study is that PI may have potential predictive value for the prognosis of post-infectious BO children. Researchers suggest that there are two mechanisms for the remission of symptoms. One is the proceeding alveolarization of lung tissues unaffected by the initial insult, the other is the increased lumen area following the global airway development. From this respect, sufficient blood supply to the lung is a precondition for the alleviation. And this may be the reason patients with worse perfusion had persistent symptoms. Based on this, we suggest that more attention should be paid to patients with severe impaired perfusion, and more aggressive treatment may be carried out in these children who may have a poor prognosis. On the other hand, as perfusion may play an important role in prognosis, we propose patients may also benefit from treatments improving pulmonary perfusion.

One intriguing finding from our preliminary study is the V/Q scan pattern in post-infectious BO children. Previous studies only reported a matched ventilation-perfusion defects with scintigraphic imaging in post-infectious BO patients [11]. However, we describe, for the first time, that there were two other patterns in children with post-infectious BO: the mismatched perfusion pattern and mismatched ventilation pattern. We speculate that these three V/Q patterns may reflect different pathological status of the ongoing inflammatory process. It is accepted that in the acute phase, inflammation of the airway will cause hypoxic vasoconstriction, and this inflammatory process may also influence the adjacent vessels, leading to vascular remodeling in the ensuing chronic phase [21]. So in the early phase of post-infectious BO, the impairment of perfusion may be less severe than that of ventilation, thus presenting with a mismatched perfusion pattern on V/Q scan. But as the inflammation progresses, the perfusion will gradually decrease, and finally result in a defective perfusion on V/Q scan. Regarding ventilation, profound inflammation leads to two types of airway impairment which are stenosis and complete obliteration. For the former, the ventilation tracer Tc-99m DTPA can spread through the narrowing airways and lead to decreased ventilation on V/Q scan. Because the corresponding perfusion may have already been destroyed at this moment, a mismatched ventilation pattern will be observed. As for the latter, Tc-99m DTPA could not pass through the completely obstructed airway and will cause a matched ventilation-perfusion pattern. Based on the pathological process, we conclude that perfusion impairment is secondary to that of ventilation, and may reflect a more severe scenario. Moreover, mismatched perfusion pattern may be a sign of the early stage of post-infectious BO, followed by matched V/Q pattern and mismatched ventilation pattern.

There were some limitations to this preliminary study. First, the sample size was small in this study, investigations involving more post-infectious BO children is warranted to further validate the clinical utility and prognostic value of V/Q scan. Second, we used planar static V/Q scan but not single-photon emission computed tomographic (SPECT) V/Q scan, the latter is now being increasingly used in respiratory researches, which is reported have the advantage in the separation of lung regions in a more detailed way, and can provide more specific information of the disease [22]. Third, the interval between V/Q scan and pulmonary function tests was set as one week, more accurate results may be obtained if the timespan was shorter. Fourthly, subjectivity existed in disease severity grading and this may lead to subjective bias. Finally, no repeated V/Q scan was carried out in our patients, we believe that repeated V/Q scan will be helpful for objectively evaluating lung function improvements.

## Conclusion

For children with post-infectious BO, the present study preliminarily indicated that the degree of ventilation and perfusion impairments evaluated by V/Q scan may be used to assess disease severity, and may be predictive of patient's outcome.
